# Educational quality of YouTube cartoon videos on children’s tooth brushing: a cross-sectional content analysis

**DOI:** 10.7717/peerj.21730

**Published:** 2026-09-18

**Authors:** İsmail Cihangir

**Affiliations:** Faculty of Dentistry, Yozgat Bozok University, Yozgat, Turkey

**Keywords:** YouTube, Cartoon videos, Children, Oral hygiene, Content analysis

## Abstract

**Background:**

YouTube is one of the most widely accessed digital platforms for health-related information, particularly among children exposed to animated content. However, the educational quality of cartoon-based videos on children’s tooth brushing remains uncertain. This study evaluated the educational value and content characteristics of YouTube cartoon videos related to children’s tooth brushing.

**Methods:**

A cross-sectional content analysis was conducted on the first 200 videos retrieved on December 28, 2025, using the keywords “children,” “tooth brushing,” and “cartoon.” After applying inclusion and exclusion criteria, 109 videos were included. Educational quality was assessed using previously published criteria and categorized into four levels. Inter-rater reliability was calculated using the intraclass correlation coefficient (ICC). Associations between educational score and video characteristics were examined using Spearman correlation and ordinal logistic regression.

**Results:**

Most videos were classified as slightly useful, and only one video (0.9%) was categorized as highly useful. The mean educational score was 2.04 ± 1.37. Tooth brushing demonstrations and daily brushing frequency were commonly presented; however, essential preventive topics such as appropriate toothpaste amount, parental supervision, floss use, and recommended starting age were frequently absent. Inter-rater reliability was good (ICC = 0.76). A weak but significant negative correlation was observed between video duration and educational score (*r* =  − 0.195, *p* = 0.042). Ordinal logistic regression showed that longer video duration was independently associated with lower odds of higher educational quality (OR = 0.33, 95% CI [0.11–0.99], *p* = 0.048). No significant associations were found between educational score and views, likes, interaction index, or upload duration. Cartoon-based YouTube videos on children’s tooth brushing demonstrate limited educational quality despite high accessibility. Popularity metrics do not reflect educational value, underscoring the need for evidence-based, structured digital oral health content for children.

## Introduction

Dental caries, the most prevalent oral health condition worldwide, begins in childhood and affects a substantial proportion of the population. The high cost of restorative treatments has led to a global shift toward preventive strategies. Particularly in developing countries, limited access to dental care and the increasing demand for health services have positioned oral health as a significant public health concern (Alwadi et al., 2025; Aksoy & Topsakal, 2022).

In children, oral health influences not only growth and development but also nutrition, mastication, speech, and self-confidence (Joufi, Claiborne & Shuman, 2021). Severe dental caries have been associated with school absenteeism, learning difficulties, and sleep disturbances, ultimately impairing quality of life. For this reason, preventive interventions should be initiated at an early age (Alwadi et al., 2025; Aksoy & Topsakal, 2022; Messina et al., 2024).

Parents, teachers, and pediatric dentists play critical roles in establishing preventive oral health behaviors (Joufi, Claiborne & Shuman, 2021). Although education provided by pediatric dentists is effective, knowledge retention may decline over time in the absence of reinforcement (Apel et al., 2025). Digital platforms offer repeatable and easily accessible content that may support behavior change and partially compensate for this gap. Beyond providing information, digital media can also influence children’s learning, attitudes, and everyday habits through repeated audiovisual exposure, thereby contributing to their education and behavioral development. However, the accuracy and scientific reliability of online content remain a concern (Alwadi et al., 2024; Ozduran & Büyükçoban, 2022).

Nevertheless, the educational influence of social media is constrained by its open and user-generated nature, variable source credibility, and the absence of systematic pre-publication quality control. These platform-related characteristics may limit the consistency, accuracy, and continuity of health messages delivered to children (Aksoy & Topsakal, 2022; Uzel et al., 2023).

Only a limited number of studies have examined digital oral hygiene content directed at children. Existing research has largely focused on parent-oriented informational videos addressing early childhood caries, fluoride. In contrast, the contribution of cartoons and animated content—media formats to which children are exposed more frequently in daily life—to oral hygiene awareness has not been adequately investigated (ElKarmi et al., 2017; Basch et al., 2018).

Given the increasing exposure of children to animated digital content and the potential influence of such media on health behaviors, evaluating the educational quality of cartoon-based oral hygiene videos has become necessary. However, the scientific accuracy and preventive value of these materials remain unclear. The aim of this study was to evaluate the educational contribution of cartoon-based content on YouTube, a platform widely used by children, to oral hygiene awareness. By identifying the strengths and deficiencies of such content, the study seeks to inform evidence-based guidance for children and parents and to support the promotion of oral health awareness at the community level.

## Materials & Methods

### Video search date and keywords

This study is based solely on the analysis of publicly accessible YouTube content and therefore did not require ethics committee approval. In accordance with institutional guidelines, ethical approval was not obtained as no individual data were collected and no intervention was performed.

The videos were searched on December 28, 2025, on the YouTube platform using the keywords “child,” “tooth brushing,” and “cartoon,” with the default “relevance” sorting filter applied. The search was conducted after clearing the browser history and without logging into a user account. The search date, keywords, and filtering method were clearly specified to ensure the reproducibility of the study.

Previous similar studies based on YouTube content analysis have examined the first 200 videos and reported that 95% of users interact with videos listed on the first page of search results (Desai et al., 2013; Nason, Donnelly & Duncan, 2016; Duman, 2020). Accordingly, the first 200 listed videos were included in the analysis.

### Inclusion criteria

• Being among the first 200 videos listed;
• providing informative content on tooth brushing for children;
• being published in Turkish.

### Exclusion criteria

• Videos not in Turkish;
• videos with poor audio quality or duplicate content;
• dental education videos intended for adults;
• videos containing advertisements or product promotions;
• videos longer than 15 min, as viewer engagement is generally reported to be short in duration (Nason, Donnelly & Duncan, 2016).

For each included video, the URL address, number of views, video duration (minutes), time elapsed since upload (days), and number of likes were recorded. To determine the level of engagement, the interaction index (number of likes/total number of views ×100) and the viewing rate (total number of views/number of days since upload ×100) were calculated using a method similar to that described by ElKarmi et al. (2017).

### Scoring criteria

**Figure 1:**
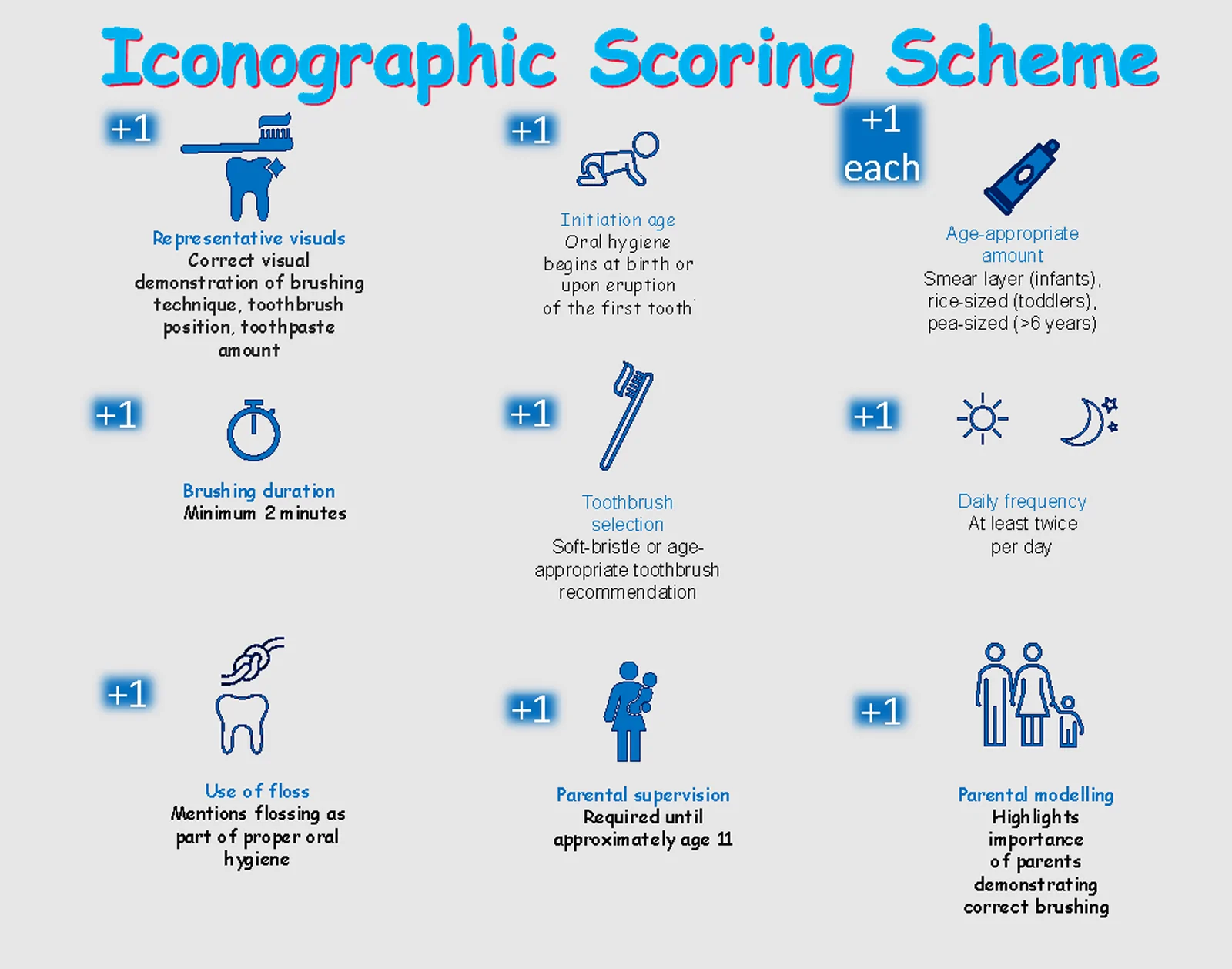
Usefulness criteria applied for the evaluation of the videos. The educational usefulness criteria used to evaluate the analyzed YouTube cartoon videos. The scoring system was adapted from previously published content analysis methodology. Videos were assigned a total score ranging from 0 to 11 based on the presence of predefined educational elements related to children’s tooth brushing. According to the total score, videos were categorized as not beneficial (0), slightly beneficial (1–3), moderately beneficial (4–7), or very beneficial (8–11).

The educational evaluation criteria used in this study were developed based on measures employed in previously published YouTube content analysis research. The maximum total score was determined as 11 (Desai et al., 2013). Accordingly, videos were classified as “not useful” (0 points), “slightly useful” (1–3 points), “moderately useful” (4–7 points), and “highly useful” (8–11 points) (Fig. 1). Prior to the evaluation process, a calibration exercise was conducted among the researchers to standardize the scoring criteria. Care was taken to ensure consistency in the application of the criteria.

To determine the reliability of the evaluation process, 40 randomly selected videos were independently re-scored by a second researcher. The second researcher evaluated the videos independently without access to the first evaluator’s scores. The intraclass correlation coefficient (ICC) for absolute agreement based on a two-way random-effects model was calculated as 0.76, indicating good agreement between the evaluators.

### Statistical analysis

Statistical analyses were performed using SPSS version 25.0 (IBM Corp., Armonk, NY, USA). Categorical variables were summarized as frequencies and percentages, while continuous variables were summarized using median, minimum, and maximum values. The distribution characteristics of continuous variables were assessed using the Shapiro–Wilk normality test. Spearman’s rho correlation analysis was conducted to examine relationships between variables. The Kruskal–Wallis test was used for group comparisons according to usefulness levels, and the Mann–Whitney U test was applied for pairwise comparisons when significant differences were identified. Ordinal logistic regression analysis was performed to determine the factors affecting educational usefulness levels. The proportional odds assumption of the model was tested. A *p*-value of 0.05 was considered statistically significant for all analyses.

## Results

**Figure 2:**
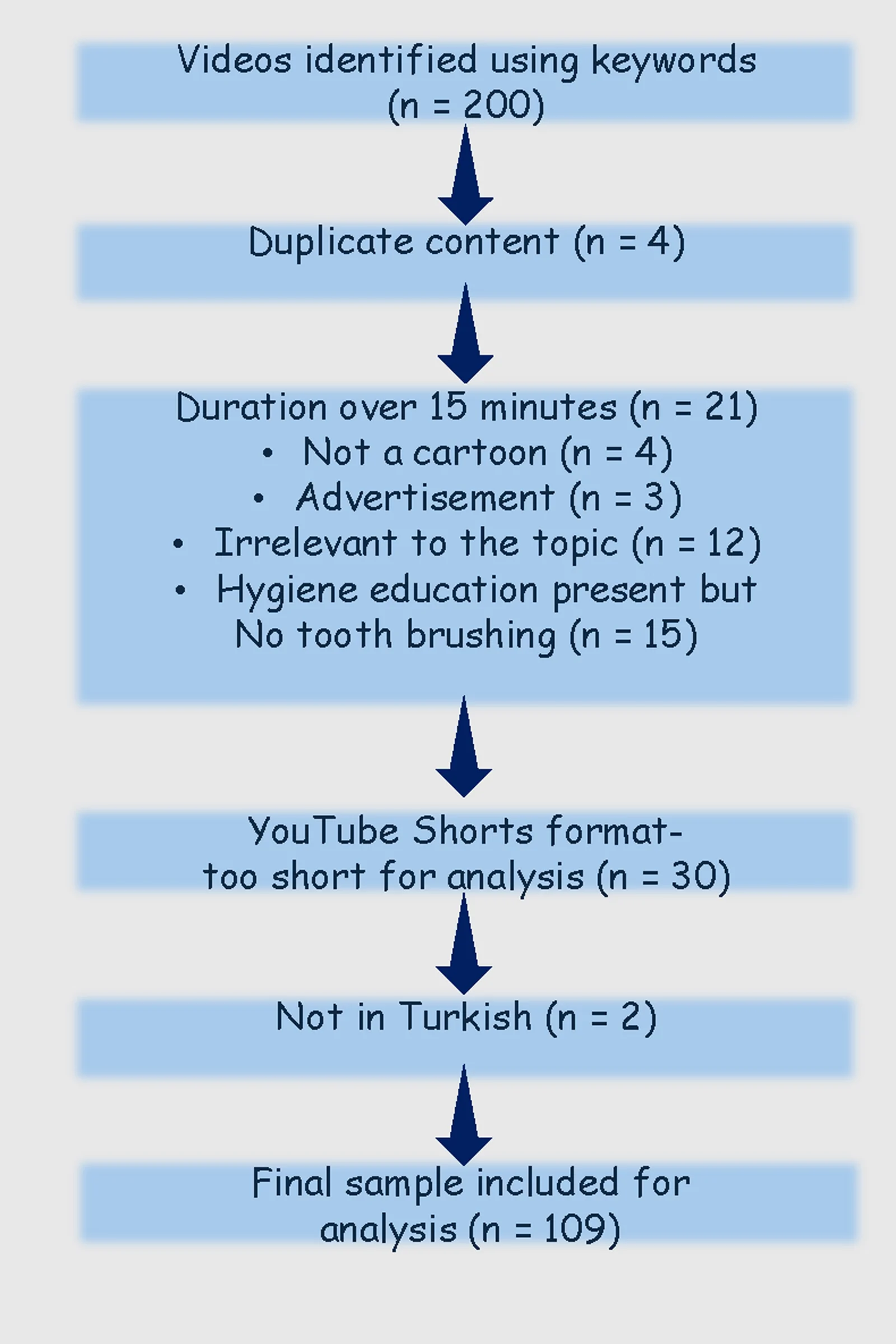
Flow diagram illustrating the identification, screening, and exclusion of videos. Flow diagram of the video selection process. A total of 200 videos were initially identified using predefined keywords. After removal of duplicate content (*n* = 4), videos exceeding 15 min (*n* = 21), non-cartoon videos (*n* = 4), advertisements (*n* = 3), videos irrelevant to the topic (*n* = 12), hygiene-related content without tooth brushing information (*n* = 15), YouTube Shorts format videos considered too brief for analysis (*n* = 30), and non-Turkish videos (*n* = 2), a final sample of 109 videos was included in the analysis.

The first 200 YouTube videos ranked by relevance using the specified keywords were screened. Of the initial 200 videos identified, duplicate videos, those not in cartoon format, and those not containing tooth brushing–related content were excluded. A total of 109 videos were included in the final analysis (Fig. 2).

**Figure 3:**
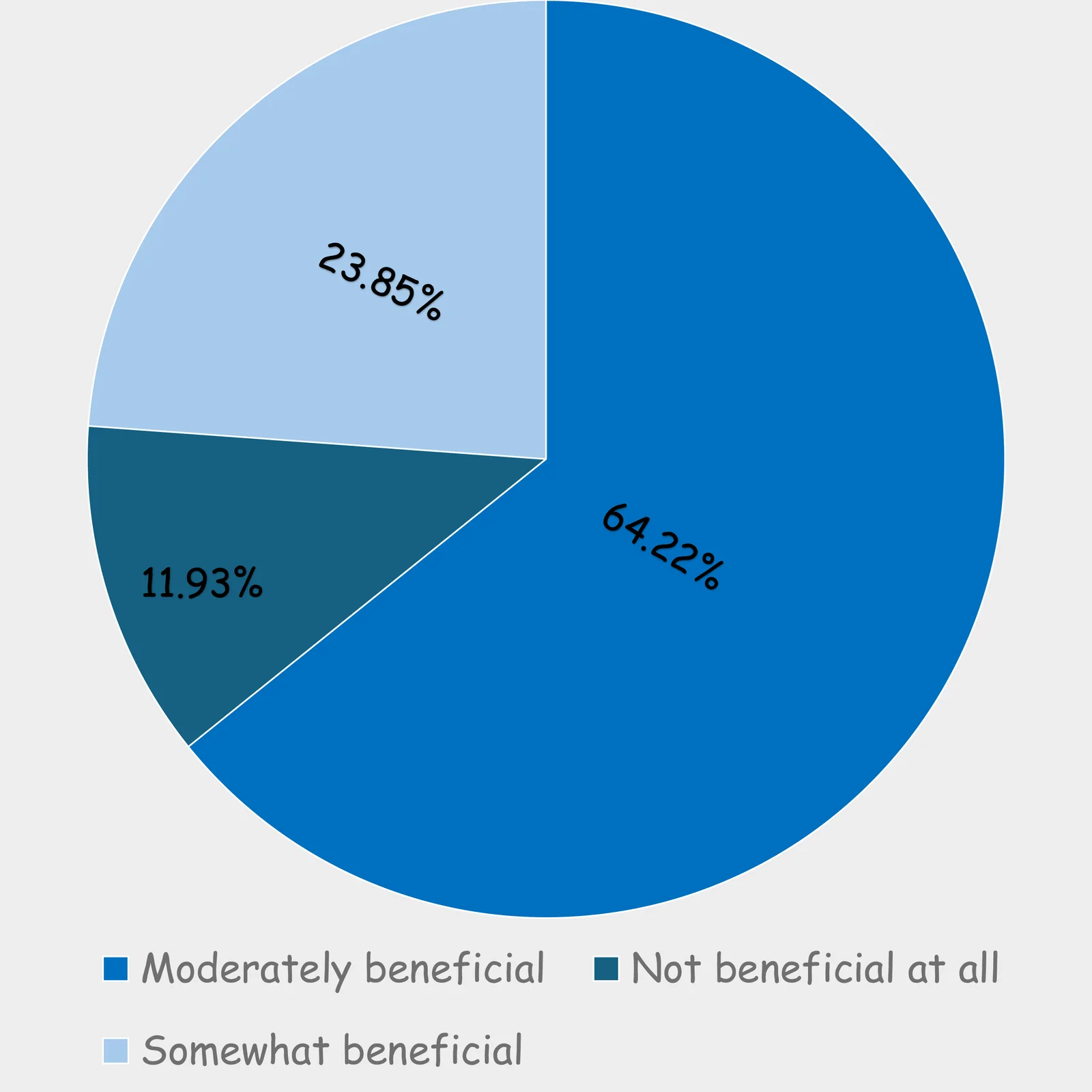
Educational value distribution of analyzed cartoon videos. Pie chart illustrating the distribution of educational usefulness levels among the 109 analyzed cartoon videos. The majority of videos were classified as moderately beneficial (64.22%), followed by somewhat beneficial (23.85%) and not beneficial at all (11.93%). No videos met the criteria for the highest educational usefulness category.

The selected videos were evaluated in terms of content and analyzed based on usefulness scores. The distribution of educational value categories is presented in Fig. 3.

Descriptive statistical data and engagement parameters of the videos are presented in Table 1. The median time since upload was 730 days (14–4,380). The mean interaction index was 4.02 ± 29.9, and the median value was 0.24 (0–296.8).

**Table 1:** Descriptive statistics and engagement parameters of analyzed YouTube cartoon videos on children’s tooth brushing. Descriptive statistics of the included videos (*n* = 109). Continuous variables are summarized as mean ± standard deviation and median (minimum–maximum). The interaction index was calculated as (number of likes/total number of views × 100), and the viewing rate was calculated as (total number of views/days since upload × 100).

Variable	Mean ± SD	Median (Min–Max)
Number of views	7,204,899.8 ± 17,747,137.7	724,000 (61–120,000,000)
Video duration (min)	5.39 ± 6.7	3.05 (1.05–57)
Days since upload	1,309.6 ± 1,329.0	730 (14–4,380)
Number of likes	1,534.14 ± 4,133.4	901 (0–254,000)
Interaction index	4.02 ± 29.9	0.24 (0–296.8)
Viewing rate	553,739.4 ± 1,775,361.7	63,926.9 (18.5–12,602,739.7)

In most videos, tooth brushing visuals and information regarding daily brushing frequency were present. However, content related to the recommended age to start brushing, use of dental floss, parental supervision, and age-appropriate toothpaste amount was less frequently included. The mean educational score was 2.04 ± 1.37, and the majority of videos were classified as “slightly useful.” Only one video (0.9%) was categorized as “highly useful” (Table 2).

**Table 2:** Content characteristics and overall educational value scores of the videos. Frequencies and percentages of content characteristics identified in the analyzed YouTube cartoon videos (*n* = 109). Educational usefulness was categorized according to the predefined scoring system (0 = not beneficial at all; 1–3 = slightly beneficial; 4–7 = moderately beneficial; 8–11 = highly beneficial). Percentages are calculated based on the total number of included videos.

Category	Yes (n, %)	No (n, %)
Representative visuals in videos	86 (78.9%)	23 (21.1%)
Toothbrushing initiation age	–	109 (100.0%)
Toothpaste amount by age	7 (6.4%)	102 (93.6%)
Toothbrush selection	32 (29.4%)	77 (70.6%)
Brushing duration	13 (11.9%)	96 (88.1%)
Daily brushing frequency	64 (58.7%)	45 (41.3%)
Use of dental floss	5 (4.6%)	104 (95.4%)
Parental supervision	10 (9.2%)	99 (90.8%)
Parental motivation for brushing	5 (4.6%)	104 (95.4%)
Overall score—Not beneficial at all	13 (11.9%)	–
Overall score—Somewhat beneficial	70 (64.2%)	–
Overall score—Moderately beneficial	25 (22.9%)	–
Overall score –Very beneficial	1 (0.9%)	–

The distribution of content characteristics according to usefulness levels is presented in Table 3. Although parental supervision, age-appropriate toothpaste amount, and daily brushing frequency appeared more frequently in higher usefulness categories, the differences between groups were not statistically significant (parental supervision: *p* = 0.363; age-appropriate toothpaste amount: *p* = 0.476; daily brushing frequency: *p* = 0.075).

**Table 3:** Distribution of content characteristics by perceived usefulness level. Values are presented as percentages within each usefulness category (Low usefulness, *n* = 83; Moderate usefulness, *n* = 25). *P*-values were calculated using Pearson’s chi-square test (or Fisher’s exact test where appropriate) to compare the distribution of content characteristics between usefulness groups. Statistically significant differences are indicated by exact *p*-values.

Content characteristic	Low usefulness (*n* = 83)	Moderate usefulness (*n* = 25)	*p*-value
Presence of representative visuals	62.7%	100.0%	0.0001
Toothbrush selection	22.9%	64.0%	0.0001
Brushing duration	7.2%	36.0%	0.0001
Brushing frequency	51.8%	84.0%	0.0090
Use of dental floss	2.4%	16.0%	0.0310
Parental supervision	6.0%	28.0%	0.0090
Motivational element for parents	2.4%	16.0%	0.0310

Spearman correlation analysis revealed a weak but statistically significant negative correlation between video duration and educational score. (Spearman’s *ρ* = −0.195, *p* = 0.042) No statistically significant correlations were found between educational score and number of views (*r* = −0.128, *p* = 0.185), number of likes (*r* = −0.121, *p* = 0.211), or days since upload (*r* = 0.014, *p* = 0.884).

Ordinal logistic regression analysis demonstrated that video duration was an independent and statistically significant predictor of educational usefulness level (OR = 0.33, 95% CI [0.11–0.99], Wald *p* = 0.048). Number of views (*p* = 0.346), number of likes (*p* = 0.857), and days since upload (*p* = 0.161) were not statistically significant predictors (Table 4).

**Table 4:** Association between video characteristics and educational score based on Spearman’s correlation and ordinal logistic regression analyses. Spearman’s rho correlation coefficients (r) and corresponding exact *p*-values are presented for the association between video characteristics and total educational score. Adjusted odds ratios (ORs) with 95% confidence intervals (CI) were obtained from ordinal logistic regression analysis. A *p*-value < 0.05 was considered statistically significant.

Variable	Spearman’s rho (r)	*p*-value	Adjusted OR	95% CI	*p*-value
Video duration	−0.195	0.042	0.33	0.11–0.99	0.048
Number of views	−0.128	0.185	0.81	0.53–1.25	0.346
Number of likes	−0.121	0.211	0.96	0.59–1.55	0.857
Days since upload	0.014	0.884	1.81	0.79–4.18	0.161

## Discussion

With the rapid advancement of technology, online platforms have become an important source for obtaining health-related information. Among these platforms, YouTube is one of the most frequently visited websites worldwide and plays a prominent role in delivering health information to children through audiovisual content. However, the findings of the present study indicate that, since content can be uploaded without being subject to standardized quality control or professional oversight, incomplete or inaccurate oral health information may be disseminated (Ozduran & Büyükçoban, 2022; Özdede & Peker, 2020).

The shortcomings identified in the present study may therefore reflect not only the content of individual videos but also the structural characteristics of YouTube as a medium. Because health-related videos can be uploaded without professional review or standardized educational requirements (Uzel et al., 2023), the information presented may be fragmented, inconsistent, or incomplete (Aksoy & Topsakal, 2022). Moreover, content visibility and popularity do not necessarily correspond to scientific accuracy or educational value, which may limit the platform’s ability to exert a coherent and sustained influence on public health. Addressing these limitations requires greater collaboration among dental professionals, health educators, and content creators, together with the development of evidence-based and age-appropriate digital materials (Uzel et al., 2023).

The educational value of content presented on YouTube has previously been evaluated in various dental topics, including early childhood caries, oral hygiene education, fluoride applications, and dental trauma (ElKarmi et al., 2017; Duman, 2020; Egil & Altan Şallı, 2020; Hutchison et al., 2020). Nevertheless, studies specifically focusing on cartoon-format videos, to which children are more frequently exposed in digital environments, remain limited. Animation-based content has been reported to increase children’s attention span, support visual learning, and promote behavioral change. In this context, the present study aimed to systematically examine the content of cartoon videos providing information about tooth brushing and to more clearly demonstrate the quality of digital oral hygiene education directed at children.

In the present study, most of the evaluated cartoon-based videos demonstrated a moderate level of usefulness, while the proportion of videos reaching the high usefulness category was limited. This finding suggests that although YouTube may serve as a potential tool for oral hygiene education in children, content quality does not exhibit a homogeneous structure. Similarly, Alwadi et al. (2025), in their evaluation of Arabic-language parental education videos, reported that the majority of videos were classified as moderate quality. Likewise, Aksoy & Topsakal (2022) indicated that pediatric oral hygiene videos contained content deficiencies, although the distribution of quality was variable and the platform possessed educational potential. Duman (2020) and Ince Kuka & Gursoy (2024) evaluated oral hygiene content on YouTube, while Tozar & Yapıcı Yavuz (2021) examined dental trauma content, and reported that the majority of videos demonstrated low to moderate usefulness. Additionally, previous studies have shown that the proportion of highly useful videos is considerably low (Narain et al., 2023; Altan Şalli & Egil, 2020). Taken together, these findings suggest that although digital oral hygiene content possesses a certain educational value, videos providing high scientific accuracy and comprehensive information remain limited. The absence of professional oversight in content uploading and the lack of standardized monitoring mechanisms may explain the variability in quality levels.

Previous studies have reported a positive association between content quality and viewer engagement. Aksoy & Topsakal (2022) demonstrated that videos with richer content had higher numbers of likes, view rates, video duration, and interaction index scores compared to videos with weaker content. Alwadi et al. (2025) suggested that higher-quality videos tend to attract greater viewer interest, although engagement cannot be explained solely by quality. Similarly, Ince Kuka & Gursoy (2024) reported that useful videos were significantly longer and had higher interaction index scores.

In contrast, the present study identified a weak but statistically significant negative correlation between video duration and educational score (*r* = −0.195; *p* = 0.042). Furthermore, ordinal logistic regression analysis indicated that video duration significantly influenced educational level, and increasing duration reduced the likelihood of being categorized in a higher usefulness level (OR = 0.33, 95% CI [0.11–0.99], *p* = 0.048). Duman (2020) also emphasized that no strong and consistent positive association was observed between video duration and quality in pediatric oral hygiene content, highlighting the heterogeneous nature of quality. Taken together, these findings suggest that in animation-format videos targeting children, longer duration does not necessarily increase educational value; rather, pedagogical suitability and attention span may be more influential factors.

Moreover, the absence of a significant relationship between educational score and view count, number of likes, or engagement metrics indicates that popularity may not reliably reflect content quality. Similarly, Narain et al. (2023) reported that videos lacking reliability may receive higher engagement due to attention-grabbing presentation features. This supports the notion that visibility on digital platforms does not always correspond to scientific accuracy or pedagogical adequacy (Mudiyanselage, Saini & Coyne, 2024).

In the present study, most cartoon-based videos included tooth brushing visuals and information regarding daily brushing frequency; however, key preventive oral health topics such as the recommended age to initiate brushing, use of dental floss, parental supervision, and age-appropriate toothpaste amount were addressed at limited levels. The low mean educational score and the presence of only one video classified as “highly useful” indicate insufficient pedagogical comprehensiveness. In contrast, Duman’s evaluation of parent-focused informational videos reported higher inclusion rates of certain fundamental content elements, although overall quality remained heterogeneous (Duman, 2020). This difference may be explained by the nature of the content format. Informational and parent-oriented videos may contain more detailed, guideline-based messages, whereas cartoon and animation formats primarily focus on visual modeling of behavior and present information in a simplified manner. Therefore, although cartoon content may be attention-grabbing and effective for behavioral modeling, it appears more limited in delivering comprehensive and structured oral hygiene information recommended by scientific guidelines.

These findings suggest that although cartoon-based digital content for children offers potential advantages in terms of engagement and behavioral modeling, it does not sufficiently meet the comprehensive and structured oral hygiene education criteria recommended in scientific guidelines. The limited coverage of key topics such as parental supervision, early initiation of brushing, and supportive preventive practices indicates that digital materials should be carefully evaluated before being used as direct references in clinical patient education. Given that dental hygienists play a crucial role in individual and community oral health education, their active involvement in the development of evidence-based and age-appropriate digital content is essential. Accordingly, the development of child-focused digital oral hygiene materials in accordance with guideline-based standards may contribute to the promotion of sustainable oral health behaviors.

## Conclusions

This study evaluated the educational adequacy of cartoon-based YouTube videos on children’s tooth brushing and found that most videos demonstrated only moderate usefulness, while highly useful content was limited. Video duration was the only independent factor associated with educational level, with longer videos being less likely to fall into higher usefulness categories. In contrast, popularity metrics such as views and likes were not reliable indicators of educational quality. Key preventive topics, including parental supervision, initiation age, and age-appropriate toothpaste use, were insufficiently addressed. These findings contribute to the growing body of evidence indicating variability in the quality of online oral health information. However, the cross-sectional design and platform-specific analysis limit causal interpretation and generalizability. Future research should explore the development and evaluation of guideline-based, age-appropriate digital oral health materials and assess their effectiveness in promoting sustained behavioral change.

##  Supplemental Information

10.7717/peerj.21730/supp-1 Supplemental Information 1 List of analyzed YouTube video URLs URLs of the 109 YouTube cartoon videos that met the inclusion criteria and were analyzed in the study. Each link corresponds to a unique video included in the final sample. Videos were identified using predefined keywords and screened according to the inclusion and exclusion criteria described in the Methods section.

10.7717/peerj.21730/supp-2 Supplemental Information 2 Raw dataset of analyzed YouTube cartoon videos on children’s tooth brushing (n = 109) Raw variables collected from the 109 YouTube cartoon videos included in the final analysis. Variables include video URL, number of views, video duration (minutes), days since upload, number of likes, interaction index, viewing rate, educational usefulness score, and content characteristics coded as binary variables (1 = present, 0 = absent).
All data were recorded on the search date specified in the Methods section. The dataset is provided to ensure transparency and reproducibility of the statistical analyses reported in the manuscript.

10.7717/peerj.21730/supp-3 Supplemental Information 3 STROBE Checklist

## Additional Information and Declarations

### Data Availability

The following information was supplied regarding data availability: The data and complete list of YouTube videos are available in the Supplemental Files.
