# Supplementary material for: Educational quality of YouTube cartoon videos on children’s tooth brushing: a cross-sectional content analysis
Source: PeerJ. 2026 Sep 18;14:e21730. doi: 10.7717/peerj.21730 (PMC13592277; doi:10.7717/peerj.21730)
Supplement: Supplementary file 1 [file peerj-14-21730-s001.docx]

1. <https://www.youtube.com/watch?v=WCvjbljKjLs&list=RDWCvjbljKjLs&start_radio=1>
2. <https://www.youtube.com/watch?v=4MAVccl_wd4&list=RD4MAVccl_wd4&start_radio=1>
3. <https://www.youtube.com/watch?v=WlbqeprY8kQ&list=RDWlbqeprY8kQ&start_radio=1>
4. <https://www.youtube.com/watch?v=09hhBTVj7RE&list=RD09hhBTVj7RE&start_radio=1>
5. <https://www.youtube.com/watch?v=NtWtwhKj5Ms&list=RDNtWtwhKj5Ms&start_radio=1>
6. <https://www.youtube.com/watch?v=f25qwpKIXwE&list=RDf25qwpKIXwE&start_radio=1>
7. <https://www.youtube.com/watch?v=MbxPgjcdQUc&list=RDMbxPgjcdQUc&start_radio=1>
8. <https://www.youtube.com/watch?v=7x3fkY7XEC4&list=RD7x3fkY7XEC4&start_radio=1>
9. <https://www.youtube.com/watch?v=C2XJFULBZ2Y&list=RDC2XJFULBZ2Y&start_radio=1>
10. <https://www.youtube.com/watch?v=dwdYzcvHNH0>
11. <https://www.youtube.com/watch?v=F1HAF6azCY8>
12. <https://www.youtube.com/watch?v=Mgo_C8IgyKA&list=RDMgo_C8IgyKA&start_radio=1>
13. <https://www.youtube.com/watch?v=hfy7tQXIvNg&list=RDhfy7tQXIvNg&start_radio=1>
14. <https://www.youtube.com/watch?v=1enaouQuphM&list=RD1enaouQuphM&start_radio=1>
15. <https://www.youtube.com/watch?v=n6stjFvAfZQ&list=RDn6stjFvAfZQ&start_radio=1>
16. <https://www.youtube.com/watch?v=mGBBgYqkQ-M&list=RDmGBBgYqkQ-M&start_radio=1>
17. <https://www.youtube.com/watch?v=0_iLRyZIcY4&list=RD0_iLRyZIcY4&start_radio=1>
18. <https://www.youtube.com/watch?v=B86MT8EejgQ>
19. <https://www.youtube.com/watch?v=lx_lPYhak98&list=RDlx_lPYhak98&start_radio=1>
20. <https://www.youtube.com/watch?v=15KRxd54BSE>
21. <https://www.youtube.com/watch?v=SctfTsLcygs>
22. <https://www.youtube.com/watch?v=ZUmKmvgQFB4&list=RDZUmKmvgQFB4&start_radio=1>
23. <https://www.youtube.com/watch?v=do_kXZBRSxI&list=RDdo_kXZBRSxI&start_radio=1>
24. <https://www.youtube.com/watch?v=UCUYr_ZnNWA&list=RDUCUYr_ZnNWA&start_radio=1>
25. <https://www.youtube.com/watch?v=L5USogGeMb8&list=RDL5USogGeMb8&start_radio=1>
26. <https://www.youtube.com/watch?v=bkgHMLubcpw&list=RDbkgHMLubcpw&start_radio=1>
27. <https://www.youtube.com/watch?v=GYfTrThsqUE>
28. <https://www.youtube.com/watch?v=tGdcqowT-Q8&list=RDtGdcqowT-Q8&start_radio=1>
29. <https://www.youtube.com/watch?v=XCY9Oun3IdQ&list=RDXCY9Oun3IdQ&start_radio=1>
30. <https://www.youtube.com/watch?v=Ag8a0q11JUs>
31. <https://www.youtube.com/watch?v=UAQ6LpX6Fh0&list=RDUAQ6LpX6Fh0&start_radio=1>
32. <https://www.youtube.com/watch?v=UKWIU8JGCJg>
33. <https://www.youtube.com/watch?v=_eBFSDY0t5o>
34. <https://www.youtube.com/watch?v=SDx_fLmB3zw>
35. <https://www.youtube.com/watch?v=7p3KpjpYQ7g>
36. <https://www.youtube.com/watch?v=EILHSyXFpsQ>
37. <https://www.youtube.com/watch?v=jleLkaGK57s>
38. <https://www.youtube.com/watch?v=lD4X_xj8lno&list=RDlD4X_xj8lno&start_radio=1>
39. <https://www.youtube.com/watch?v=UMm6vc4Lus0&list=RDUMm6vc4Lus0&start_radio=1>
40. <https://www.youtube.com/watch?v=eCwvnBosoj0&list=RDeCwvnBosoj0&start_radio=1>
41. <https://www.youtube.com/watch?v=useHA-DmKq8&list=RDuseHA-DmKq8&start_radio=1>
42. <https://www.youtube.com/watch?v=7hmBkw25--U&list=RD7hmBkw25--U&start_radio=1>
43. <https://www.youtube.com/watch?v=bVD6ykxASHo>
44. <https://www.youtube.com/watch?v=106MTsYiuKQ&list=RD106MTsYiuKQ&start_radio=1>
45. <https://www.youtube.com/watch?v=hfy7tQXIvNg&list=RDhfy7tQXIvNg&start_radio=1>
46. <https://www.youtube.com/watch?v=sxeSGJIcrNg&list=RDsxeSGJIcrNg&start_radio=1>
47. <https://www.youtube.com/watch?v=PwjEn2c_Prc>
48. <https://www.youtube.com/watch?v=EBUi5zgQrnE>
49. <https://www.youtube.com/watch?v=_eBFSDY0t5o>
50. <https://www.youtube.com/watch?v=e5iMQEnQyV4>
51. <https://www.youtube.com/watch?v=HeCzXMHX3_8&list=RDHeCzXMHX3_8&start_radio=1>
52. <https://www.youtube.com/watch?v=n_Z9n2ocWDQ>
53. <https://www.youtube.com/watch?v=ZQn5au_aMZw>
54. <https://www.youtube.com/watch?v=E5SbMvcTEOU&list=RDE5SbMvcTEOU&start_radio=1>
55. <https://www.youtube.com/watch?v=f3K7VpVGU18&list=RDf3K7VpVGU18&start_radio=1>
56. <https://www.youtube.com/watch?v=rf9da9DzM_U&list=RDrf9da9DzM_U&start_radio=1>
57. <https://www.youtube.com/watch?v=YGFmDx-L_dk>
58. <https://www.youtube.com/watch?v=0_iLRyZIcY4&list=RD0_iLRyZIcY4&start_radio=1>
59. <https://www.youtube.com/watch?v=6xGouD_nAqM&list=RD6xGouD_nAqM&start_radio=1>
60. <https://www.youtube.com/watch?v=6HmFv6jzekA>
61. <https://www.youtube.com/watch?v=sxeSGJIcrNg&list=RDsxeSGJIcrNg&start_radio=1>
62. <https://www.youtube.com/watch?v=J6HfORjZ1z4&list=RDJ6HfORjZ1z4&start_radio=1>
63. <https://www.youtube.com/watch?v=vHYqR0iB4f4&list=RDvHYqR0iB4f4&start_radio=1>
64. <https://www.youtube.com/watch?v=3DEKHnfHiGc&list=RD3DEKHnfHiGc&start_radio=1>
65. <https://www.youtube.com/watch?v=pHh6eROWlys&list=RDpHh6eROWlys&start_radio=1>
66. <https://www.youtube.com/watch?v=diqRQUCoPec&list=RDdiqRQUCoPec&start_radio=1>
67. <https://www.youtube.com/watch?v=wwY6adFxnuo&list=RDwwY6adFxnuo&start_radio=1>
68. <https://www.youtube.com/watch?v=pMH7IeNQZCw&list=RDpMH7IeNQZCw&start_radio=1>
69. <https://www.youtube.com/watch?v=IGTbpn8j7MY>
70. <https://www.youtube.com/watch?v=ytaTbQ4UNKg&list=RDytaTbQ4UNKg&start_radio=1>
71. <https://www.youtube.com/watch?v=xlnOPJL1Rg8&list=RDxlnOPJL1Rg8&start_radio=1>
72. <https://www.youtube.com/watch?v=JQNCrflyYuo>
73. <https://www.youtube.com/watch?v=kV7iCtsI2aQ>
74. <https://www.youtube.com/watch?v=E5SbMvcTEOU&list=RDE5SbMvcTEOU&start_radio=1>
75. <https://www.youtube.com/watch?v=f3K7VpVGU18&list=RDf3K7VpVGU18&start_radio=1>
76. <https://www.youtube.com/watch?v=1iZMeOe_X4k&list=RD1iZMeOe_X4k&start_radio=1>
77. <https://www.youtube.com/watch?v=YGFmDx-L_dk>
78. <https://www.youtube.com/watch?v=yRY89x-hQ1Q>
79. <https://www.youtube.com/watch?v=6xGouD_nAqM&list=RD6xGouD_nAqM&start_radio=1>
80. <https://www.youtube.com/watch?v=pMH7IeNQZCw&list=RDpMH7IeNQZCw&start_radio=1>
81. <https://www.youtube.com/watch?v=DGkM2MBpOQw&list=RDDGkM2MBpOQw&start_radio=1>
82. <https://www.youtube.com/watch?v=4Q3yovo_wWQ&list=RD4Q3yovo_wWQ&start_radio=1>
83. <https://www.youtube.com/watch?v=rIoO8TjcNNM&list=RDrIoO8TjcNNM&start_radio=1>
84. <https://www.youtube.com/watch?v=shRD-g7KIDM>
85. <https://www.youtube.com/watch?v=Fk59hVMjDHU>
86. <https://www.youtube.com/watch?v=4Q3yovo_wWQ&list=RD4Q3yovo_wWQ&start_radio=1>
87. <https://www.youtube.com/watch?v=J6HfORjZ1z4&list=RDJ6HfORjZ1z4&start_radio=1>
88. <https://www.youtube.com/watch?v=bngGz856wwY>
89. <https://www.youtube.com/watch?v=3DEKHnfHiGc&list=RD3DEKHnfHiGc&start_radio=1>
90. <https://www.youtube.com/watch?v=asqLcPvlQFY>
91. <https://www.youtube.com/watch?v=ohZAjfHcCi8&list=RDohZAjfHcCi8&start_radio=1>
92. <https://www.youtube.com/watch?v=pHh6eROWlys&list=RDpHh6eROWlys&start_radio=1>
93. <https://www.youtube.com/watch?v=4d_kNKCBeik>
94. <https://www.youtube.com/watch?v=diqRQUCoPec&list=RDdiqRQUCoPec&start_radio=1>
95. <https://www.youtube.com/watch?v=Qk6xaiQns-M>
96. <https://www.youtube.com/watch?v=ytaTbQ4UNKg&list=RDytaTbQ4UNKg&start_radio=1>
97. <https://www.youtube.com/watch?v=xlnOPJL1Rg8&list=RDxlnOPJL1Rg8&start_radio=1>
98. <https://www.youtube.com/watch?v=4Q3yovo_wWQ&list=RD4Q3yovo_wWQ&start_radio=1>
99. <https://www.youtube.com/watch?v=ix6UrgP6Wxk>
100. <https://www.youtube.com/watch?v=94Mr3jwiiBo&list=RD94Mr3jwiiBo&start_radio=1>
101. <https://www.youtube.com/watch?v=JWn1lAFCH9I>
102. <https://www.youtube.com/watch?v=HeCzXMHX3_8&list=RDHeCzXMHX3_8&start_radio=1>
103. <https://www.youtube.com/watch?v=14ftkkwJr5I&list=RD14ftkkwJr5I&start_radio=1>
104. <https://www.youtube.com/watch?v=Ex0gOIGQuyg&list=RDEx0gOIGQuyg&start_radio=1>
105. <https://www.youtube.com/watch?v=8AWLDUtJTEw>
106. <https://www.youtube.com/watch?v=kNufdsWY-tU>
107. <https://www.youtube.com/watch?v=R16vr68TPvg&list=RDR16vr68TPvg&start_radio=1>
108. <https://www.youtube.com/watch?v=DGkM2MBpOQw&list=RDDGkM2MBpOQw&start_radio=1>
109. <https://www.youtube.com/watch?v=rIoO8TjcNNM&list=RDrIoO8TjcNNM&start_radio=1>
