# Supplementary material for: Educational quality of YouTube cartoon videos on children’s tooth brushing: a cross-sectional content analysis
Source: PeerJ. 2026 Sep 18;14:e21730. doi: 10.7717/peerj.21730 (PMC13592277; doi:10.7717/peerj.21730)
Supplement: Supplementary file 2 [file peerj-14-21730-s003.doc]

STROBE Statement—Checklist of items that should be included in reports of ***cross-sectional studies***

|  | Item No | Recommendation | **Explanation** |  |  |
| --- | --- | --- | --- | --- | --- |
| **Title and abstract** | 1 | (*a*) Indicate the study’s design with a commonly used term in the title or the abstract | Location in manuscript: title; abstract lines 1-3 methods lines 16-22 |  |  |
|  |  | (*b*) Provide in the abstract an informative and balanced summary of what was done and what was found | Location in manuscript: Abstract (Methods and Results) lines 11-34 |  |  |
| Introduction | | |  |  |  |
| Background/rationale | 2 | Explain the scientific background and rationale for the investigation being reported | Scientific background and rationale are described in the Introduction , including the need to evaluate the educational  quality of cartoon-based oral health videos on YouTube. Lines 37-66 |  |  |
| Objectives | 3 | State specific objectives, including any prespecified hypotheses | The specific objective of the study is stated in the final paragraph of the Introduction , describing the evaluation  of the educational quality of cartoon-based YouTube videos on children’s tooth brushing.Lines 59-66. |  |  |
| Methods | | |  |  |  |
| Study design | 4 | Present key elements of study design early in the paper | The study was designed as a cross-sectional content analysis of YouTube cartoon videos related to children’s tooth  brushing.  Location in manuscript: (Materials and Methods – first paragraph under “Video search date and keywords”)  lines 67-73 |  |  |
| Setting | 5 | Describe the setting, locations, and relevant dates, including periods of recruitment, exposure, follow-up, and data collection | The YouTube search was conducted on December 28, 2025, using predefined keywords and the default relevance  filter. Only publicly accessible Turkish-language videos were screened and included based on eligibility criteria. Location in manuscript: (Materials and Methods – “Video search date and keywords” section )  Lines 74-81 |  |  |
| Participants | 6 | (*a*) Give the eligibility criteria, and the sources and methods of selection of participants | The first 200 videos retrieved were screened according to predefined inclusion and exclusion criteria, and 109  eligible videos were included in the final analysis. Location in manuscript: (Materials and Methods – “Inclusion and exclusion criteria”; Results – flow diagram )  lines 123-127 |  |  |
| Variables | 7 | Clearly define all outcomes, exposures, predictors, potential confounders, and effect modifiers. Give diagnostic criteria, if applicable | The primary outcome variable was educational usefulness level based on a predefined scoring system (0–11 points).  Predictor variables included video duration, number of views, number of likes, upload duration, interaction index,  and viewing rate. Location in manuscript: (Materials and Methods – “Scoring criteria” and “Statistical analysis” sections )  Lines 111-122 | L |  |
| Data sources/ measurement | 8* | For each variable of interest, give sources of data and details of methods of assessment (measurement). Describe comparability of assessment methods if there is more than one group | Video characteristics were obtained directly from the YouTube interface at the time of data collection.  Educational quality was independently evaluated by two researchers using predefined criteria, and inter-rater  reliability was assessed using the intraclass correlation coefficient (ICC). Location in manuscript: (Materials and Methods – “Video search,” “Scoring criteria,” and reliability description )  Lines 93-97 and 106-110 |  |  |
| Bias | 9 | Describe any efforts to address potential sources of bias | To minimize selection bias, a predefined search strategy and standardized inclusion/exclusion criteria were applied.  To reduce assessment bias, two independent researchers evaluated all videos, and inter-rater reliability was assessed  using ICC. Location in manuscript: (Materials and Methods – “Video search date and keywords,”  “Inclusion and exclusion criteria,” and reliability description )  Lines 74-81; 106-110 |  |  |
| Study size | 10 | Explain how the study size was arrived at | The study sample consisted of the first 200 videos retrieved using predefined keywords, and 109 videos met the  eligibility criteria and were included in the final analysis. Location in manuscript: (Materials and Methods – “Video search date and keywords”; Lines 79-81  Results – flow diagram page 2 Lines 123-127 |  |  |
| Quantitative variables | 11 | Explain how quantitative variables were handled in the analyses. If applicable, describe which groupings were chosen and why | Quantitative variables (e.g., video duration, number of views, likes, upload duration) were analyzed as continuous  variables. Educational score was categorized into four ordinal levels (not useful, slightly useful, moderately useful,  highly useful) based on predefined score ranges. Location in manuscript: (Materials and Methods – “Scoring criteria” and “Statistical analysis” )  Lines 98-105; 111-122 | L | Ines |
| Statistical methods | 12 | (*a*) Describe all statistical methods, including those used to control for confounding | Data normality was assessed, and associations between educational score and video characteristics were  analyzed using Spearman correlation. Ordinal logistic regression was performed to evaluate independent  associations while controlling for potential confounders. Statistical significance was set at p < 0.05. Location in manuscript: (Materials and Methods – “Statistical analysis”)  Lines 111-122 |  |  |
|  |  | (*b*) Describe any methods used to examine subgroups and interactions | No subgroup or interaction analyses were performed in this study. Location in manuscript: (Materials and Methods – not applicable; no subgroup analysis reported) |  |  |
|  |  | (*c*) Explain how missing data were addressed | Videos with incomplete data or inaccessible content were excluded during the screening process;  therefore, no missing data were present in the final dataset. Location in manuscript: (Materials and Methods – “Inclusion and exclusion criteria”)  Lines 82-92 |  |  |
|  |  | (*d*) If applicable, describe analytical methods taking account of sampling strategy | Since a predefined search strategy was used without probabilistic sampling, no additional analytical adjustments  for sampling strategy were required. Location in manuscript: (Materials and Methods – search strategy description)  Lines 74-81 |  |  |
|  |  | (*e*) Describe any sensitivity analyses | No sensitivity analyses were conducted. Location in manuscript: (Materials and Methods – not applicable) |  |  |
| Results | | |  |  |  |
| Participants | 13* | (a) Report numbers of individuals at each stage of study—eg numbers potentially eligible, examined for eligibility, confirmed eligible, included in the study, completing follow-up, and analysed | The first 200 YouTube videos were screened. After applying the inclusion and exclusion criteria,  109 videos were included in the final analysis. Location in manuscript: Results – first paragraph + Figure 2  (flow diagram).  Lines 123-127 |  |  |
|  |  | (b) Give reasons for non-participation at each stage | Videos were excluded if they were duplicates, not in cartoon format, not related to tooth brushing, not in Turkish,  contained advertisements, had poor audio quality, or exceeded 15 minutes in duration.  Location in manuscript: Materials & Methods – Inclusion and Exclusion Criteria + Results first paragraph.  Lines 82-92 ; 123-126 |  |  |
|  |  | (c) Consider use of a flow diagram | A flow diagram (Figure 2) illustrates the video selection process.Line 127 |  |  |
| Descriptive data | 14* | (a) Give characteristics of study participants (eg demographic, clinical, social) and information on exposures and potential confounders | Descriptive characteristics of the included videos, including duration, number of views, likes, time since upload,  interaction index, and educational scores, are presented in Table 1.  Location in manuscript: Results – Descriptive statistical data paragraph + Table 1.  Lines 128-138 |  |  |
|  |  | (b) Indicate number of participants with missing data for each variable of interest | No missing data were identified for the variables analyzed. Location in manuscript: Methods – Data Collection  Lines 82-92 |  |  |
| Outcome data | 15* | Report numbers of outcome events or summary measures | The primary outcome was educational usefulness score.  The mean educational score was 2.04 ± 1.37, and only one video (0.9%) was classified as highly useful.  Distribution across usefulness categories is presented in Table 2 and Figure 3.  Location in manuscript: Results – educational score paragraph + Table 2 + Figure 3.  Lines 133-138 |  |  |
| Main results | 16 | (*a*) Give unadjusted estimates and, if applicable, confounder-adjusted estimates and their precision (eg, 95% confidence interval). Make clear which confounders were adjusted for and why they were included | Unadjusted associations between educational score and video characteristics were examined using  Spearman correlation analysis. A weak but statistically significant negative correlation was observed between video  duration and educational score (r = –0.195, p = 0.042).  Ordinal logistic regression analysis was performed to evaluate independent predictors of educational quality.  Video duration remained significantly associated with lower odds of higher educational quality  (OR = 0.33, 95% CI: 0.11–0.99, p = 0.048).  The regression model included video duration, number of views, likes, interaction index, and upload duration as  potential confounding variables.  Location in manuscript: Results – Statistical Analysis & Results section  (Correlation and Ordinal Logistic Regression paragraphs)  Lines 145-153 |  |  |
|  |  | (*b*) Report category boundaries when continuous variables were categorized | Educational usefulness scores were categorized into four predefined levels  (not useful, slightly useful, moderately useful, and highly useful) based on previously published scoring criteria.  Location in manuscript: Methods – Educational Scoring Criteria subsection  Lines 98-105 |  |  |
|  |  | (*c*) If relevant, consider translating estimates of relative risk into absolute risk for a meaningful time period | Translation of relative risk into absolute risk was not applicable due to the cross-sectional design of the study. |  |  |
| Other analyses | 17 | Report other analyses done—eg analyses of subgroups and interactions, and sensitivity analyses | Additional analyses included correlation and ordinal logistic regression models to evaluate the relationship  between video characteristics and educational quality. No subgroup or sensitivity analyses were performed.  Lines 145-153 |  |  |
| Discussion | | |  |  |  |
| Key results | 18 | Summarise key results with reference to study objectives | The key findings are summarized in the final paragraphs of the Discussion and in the Conclusions section,  directly linked to the study objective of evaluating the educational quality of cartoon-based YouTube videos on  children’s tooth brushing. the key findings are summarized in the final paragraphs of the Discussion and in the  Conclusions section, directly linked to the study objective of evaluating the educational quality of cartoon-based  YouTube videos on children’s tooth brushing.  Location in manuscript: Discussion (final paragraphs) and Conclusions  Lines 171-184 and Lines 235-243 |  |  |
| Limitations | 19 | Discuss limitations of the study, taking into account sources of potential bias or imprecision. Discuss both direction and magnitude of any potential bias | Study limitations, including the cross-sectional design, single-platform evaluation, dynamic nature of  YouTube content, and potential selection bias due to search algorithm variability, are discussed in the Conclusions  section. The possible impact of these factors on internal validity and generalisability is acknowledged.  Location in manuscript: Conclusions (limitations paragraph)  Lines 244-248 |  |  |
| Interpretation | 20 | Give a cautious overall interpretation of results considering objectives, limitations, multiplicity of analyses, results from similar studies, and other relevant evidence | A cautious interpretation of the findings is provided in the final section of the Discussion,  integrating study objectives, methodological limitations, and comparison with previous literature on digital  oral health content. Location in manuscript: Discussion (final section)  Lines 194-223 |  |  |
| Generalisability | 21 | Discuss the generalisability (external validity) of the study results | The limited external validity due to platform-specific sampling, language restrictions (if applicable),  and time-dependent search results is discussed in the Conclusions section. Location in manuscript: Conclusions  Lines 244-248 |  |  |
| Other information | | |  |  |  |
| Funding | 22 | Give the source of funding and the role of the funders for the present study and, if applicable, for the original study on which the present article is based | This study received no external funding. The authors declare that no funding body had any role in study design,  data collection, analysis, interpretation, or manuscript preparation. Location in manuscript:  Location in manuscript: Funding Statement / Declarations section lines 249 |  |  |

*Give information separately for exposed and unexposed groups.

**Note:** An Explanation and Elaboration article discusses each checklist item and gives methodological background and published examples of transparent reporting. The STROBE checklist is best used in conjunction with this article (freely available on the Web sites of PLoS Medicine at http://www.plosmedicine.org/, Annals of Internal Medicine at http://www.annals.org/, and Epidemiology at http://www.epidem.com/). Information on the STROBE Initiative is available at www.strobe-statement.org.
